# Heterologous SARS-CoV-2 Buccal Immunization with Oral Dissolving Films Generated a Strong Systemic and Mucosal Immunity in a Murine Model

**DOI:** 10.3390/vaccines13111105

**Published:** 2025-10-29

**Authors:** Tanisha Manoj Arte, Smital Patil, Emmanuel Adediran, Mahek Gulani, Amarae Ferguson, Sarthak Shah, Priyal Bagwe, Susu M. Zughaier, Martin J. D’Souza

**Affiliations:** 1 Vaccine Nanotechnology Laboratory, Center for Drug Delivery Research, College of Pharmacy, Mercer University, Atlanta, GA 30341, USA; tanisha.manoj.arte@live.mercer.edu (T.M.A.); smitalrajan.patil@live.mercer.edu (S.P.); mahekanil.gulani@live.mercer.edu (M.G.); amarae.ferguson@live.mercer.edu (A.F.); priyal.bagwe@live.mercer.edu (P.B.); 2 Department of Pharmaceutical Sciences, College of Pharmacy, Larkin University, Miami, FL 33169, USA; eadediran@larkin.edu; 3 College of Science, Health and Pharmacy, Roosevelt University, Schaumburg, IL 60173, USA; sarthak.modi.shah@live.mercer.edu; 4 College of Medicine, QU Health, Qatar University, Doha P.O. Box 2713, Qatar

**Keywords:** heterologous, microparticulate, COVID-19, delta variant, omicron variant, oral dissolving films, cross-reactivity, neutralization assay, antibodies, cellular response, autophagy, mucosal response

## Abstract

Background: In response to the emergence of immune-evasive variants of SARS-CoV-2, this study explores a novel heterologous vaccination strategy using a microparticulate formulation approach that is delivered via oral dissolving film (ODF) formulations into the buccal cavity. Heterologous administration has the potential to generate cross-reactive antibodies, which can be especially beneficial against viruses with ever-mutating variants. Moreover, the microparticulate oral dissolving film-based vaccine approach is a non-invasive vaccine delivery platform. Methods: The vaccine design incorporated whole inactivated Delta and Omicron variants of the virus, administered at prime and booster doses, respectively, effectively encapsulated in a Poly(lactic-co-glycolic) acid (PLGA) polymer matrix, and adjuvanted with Alum to enhance immune activation. Following vaccination, serum, mucosal, and tissue samples were analyzed to evaluate humoral and cellular immune responses against the model antigen, as well as other variants such as Alpha and Beta variants, to understand the cross-reactive response. Result: In vitro evaluations confirmed the vaccine’s safety and its ability to stimulate immune responses. On administering microparticulate oral dissolving films to mice, whole inactivated delta and omicron variant-specific antibodies were observed in serum samples along with neutralizing titers in terminal week. The formulated vaccine showed significant secretory IgA antibody levels in mucosal samples. Moreover, CD4^+^ and CD8a cellular responses were observed in tissue samples of spleen and lymph nodes, along with antibodies (IgG, IgA, and IgM) detected in lung supernatant samples. Humoral and cellular cross-reactive antibodies were observed in the samples. Conclusions: This approach offers a promising platform for developing next-generation vaccines capable of inducing broad immunity.

## 1. Introduction

SARS-CoV-2, the novel coronavirus responsible for COVID-19, emerged in late 2019 and rapidly escalated into a global pandemic, profoundly impacting public health and daily life. Over the years, various strains of the SARS-CoV-2 virus, for instance Alpha, Beta, Gamma, Delta, and Omicron, have emerged due to the addition and deletion of different mutations. These have led to around 1.2 million deaths in the United States and more deaths worldwide [1]. The mutations lead to differences in the transmissibility of the virus, disease severity, and also display the ability to evade immune invasion. Delta was one of the variants that led to the highest hospitalization with higher viral loads and was deadly during the 2nd wave of the COVID-19 pandemic. However, the Omicron variant that arrived later had higher transmissibility as it strongly escaped the immune system [2,3]. Currently various sub-variants of Omicron such as NB.1.8.1, KP.3, KP.3.1.1, JN.1, JN.1.18, LP.8.1, XEC, XFG, various sub-variants of Omicron, such as NB.1.8.1, KP.3, KP.3.1.1, JN.1, JN.1.18, LP.8.1, XEC, XFG, are circulating globally [4].

This virus is not only responsible for causing flu-like symptoms, but clinical manifestations include severe conditions such as acute respiratory distress syndrome (ARDS), multi-organ failure, septic shock influenced by demographic comorbidities, and genetic factors. Even after the infection is cleared, individual patients may experience post-acute complications caused by the viral tissue infection, which can impact various other systems [5,6]. Immunizing the population avoids these complications considerably. Although there are various vaccines available, their effectiveness against the evolving variants of the virus is questionable. As of now, the CDC recommends individuals receive a COVID-19 vaccine with the JN1 variant; however, the efficacy of this vaccine is yet to be tested against the latest sub-variant such as XFG (14%), NB.1.8.1 (43%), and LP.8.1 (31%) in the US [7].

Even though the pandemic is over, the world is still facing the effects of the post-COVID-19 era. Research with a new strategy will take a step towards designing safer and more versatile vaccines to combat emerging variants. In this study, we have used a heterologous prime booster microparticulate vaccine strategy. This refers to using different vaccine variants for the prime and booster doses, rather than repeating the same formulation at both doses. Such a type of strategy provides various advantages that can improve immunogenicity of vaccine formulation due to interaction of the immune system with different variants of the virus, also providing better cross-variant reactive immune response. This strategy was used in influenza vaccines earlier, which provided broader immunity across multiple strains and is now being explored for COVID-19 vaccines [8,9,10].

Mucosal vaccines can elicit strong mucosal immunity, including an IgA response and resident T and B cells in the respiratory tract, providing better protection against respiratory pathogens such as SARS-CoV-2 variants. Recent studies on the microparticulate vaccine approach have shown that vaccines coated on polymeric particles converted to the micron size can enhance immune activation [11]. The foreign nature of these particles promotes the uptake by antigen-presenting cells, thus eliciting a stronger and more durable immune response compared to traditional vaccine formulations [12,13]. In addition, using FDA-approved biodegradable and biocompatible polymer like PLGA (poly (lactic-co-glycolic acid)) is metabolized in the body to lactic and glycolic acid and safely absorbed and eliminated, thus providing an excellent safety profile, with minimal toxicity. Moreover, PLGA has a controlled release profile that enables the gradual release of antigen, which is optimal for efficient immune induction and mimics natural infection patterns [14]. We have demonstrated the immune activation and cytotoxicity assessment in vitro by antigen-presenting cells, such as dendritic cells (DCs), for whole inactivated delta and omicron microparticles of the SARS-CoV-2 virus in our previously published paper [15]. Adjuvants are also used in this study, as they are known to increase the magnitude, durability, and quality (breadth and affinity) of the immune response to vaccine antigens. The particulate nature of Alum facilitates uptake by antigen-presenting cells (APCs) such as dendritic cells and macrophages. This accelerates phagocytosis, boosts immunological memory, and leads to a more robust immune response [16,17].

Traditional intramuscular route is the painful route of administration leading to needle phobia, need of trained professionals for delivery of vaccine, and administration site injuries. Additionally, vaccine formulation requires cold-chain maintenance and aseptic handling, which limits large-scale immunization efforts, particularly in resource-limited countries. An effective alternative is delivery of vaccine through buccal route using oral dissolving films. In this study, we plan to explore a non-invasive buccal route. Buccal vaccine delivery targets the inner cheek mucosa, utilizing its relatively permeable, non-keratinized stratified epithelium to facilitate antigen transfer into systemic and mucosal immune compartments. After administration, the vaccine formulation adheres to the buccal mucosa-associated lymphoid tissue (MALT). Antigen molecules then cross the buccal epithelium by M cells and are taken up by antigen-presenting cells (APCs) in the epithelial layer underlying lamina propria. Moreover, vaccines delivered via this route can also penetrate the mucosal barrier through paracellular (between cells) and transcellular (through cells) pathways and up taken by APCs. These APCs migrate to secondary lymphoid tissues, mucosa-associated lymphoid tissue, to initiate both local and systemic immune responses [18,19].

This proof-of-concept study explores a strategy not previously reported for COVID-19 that involves polymeric microparticles (MP) as an antigen delivery platform using a heterologous strategy for delivering oral dissolving film through buccal vaccination. We formulated PLGA-based microparticulate ODFs encapsulating whole inactivated virus of SARS-CoV-2 variant Delta and Omicron as a model antigen, along with Alum as an Adjuvant MPs. We further examined the expression of major histocompatibility complex (MHC I) and MHC II molecules, along with their respective costimulatory markers, on dendritic cells after stimulation with MPs. Lastly, the in vivo immunogenic potential of vaccine MPs, with adjuvant, was assessed by measuring antibody responses following buccal administration. Cellular T-cell response in the organ sample was also analyzed post-sacrifice.

## 2. Materials and Methods

### 2.1. Materials

BEI resources provided us with heat-treated and gamma-irradiated whole inactivated virus of SARS-CoV-2 for the Delta and Omicron variants, along with other reagents such as NR-52511—Human Embryonic Kidney Cells (HEK-293T) Expressing Human Angiotensin-Converting Enzyme 2 cell Line, Wuhan-Hu-1 Spike D614G-Pseudotyped Lentivirus, and Luc2/ZsGreen. Poly(lactic-co-glycolic) acid (PLGA) 75:25 (Resomer^®^ RG 752H) was purchased from Evonik Industries. Fischer Scientific provided us with dichloromethane (DCM). Millipore Sigma (Burlington, MA, USA) supplied Span^®^ 80 trehalose dihydrate. Thermo Fischer (Waltham, MA, USA) provided the Pierce Micro BCA Assay Kit and 3-(4,5-dimethylthiazol-2-yl)-2,5-diphenyltetrazolium bromide (MTT). Kollidon F90 and Kollidon VA 64 were purchased from BASF in Houston, TX, USA. Adjuvants, such as Alhydrogel^®^ (Alum) (InvivoGen, San Diego, CA, USA) were bought from InvivoGen (San Diego, CA, USA). 3,3′,5,5′-tetramethylbenzidine (TMB) was obtained from Becton, Dickinson & Co. (Franklin Lakes, NJ, USA). Swiss Webster mice aged six to eight weeks were obtained from Charles River Laboratories (Wilmington, MA, USA). Dulbecco’s Modified Eagle’s Medium (DMEM), trypsin-EDTA solution, fetal bovine serum (FBS), and penicillin-streptomycin were obtained from the American Type Culture Collection in Manassas, VA, USA. Cell culture lysis reagent 5X was ordered from Promega Corporation, Madison, WI, USA. Polybrene transfection reagent was purchased from Millipore Corporation. HRP-tagged secondary goat anti-mouse IgM, IgG, IgA, IgG2a, and IgG1 were purchased from Invitrogen (Waltham, MA, USA). CYTO-ID^®^ Autophagy detection kit was purchased from Enzo Life Sciences (Farmingdale, NY, USA). Anti-mouse antibodies, such as fluorescein isothiocyanate (FITC)-labeled MHC I CD80, CD8a, and allophycocyanin-labeled MHC II, CD40, and CD4, used for flow cytometry analysis, were purchased from eBioscience Laboratories (San Diego, CA, USA) and BioLegend (San Diego, CA, USA).

### 2.2. Formulation of the Vaccine Microparticles and Oral Dissolving Films

Microparticles (MPs) were prepared utilizing the previously developed (*w*/*o*/*w*) double emulsion process in our lab [20]. First, the primary emulsion was generated, then the secondary emulsion. To create the primary emulsion, an aqueous phase containing 2% antigen, which is made up of the entire inactivated virus of Delta/Omicron, is mixed with an organic phase containing 2% *w*/*v* PLGA (polymer) in dichloromethane (DCM) and Span 80 as a surfactant. The mixture is then homogenized at 17,000 RPM for 2 min to produce the primary emulsion. The secondary emulsion is created by adding the original emulsion to the aqueous phase, which is composed of polyvinyl alcohol in deionized water. The mixture is homogenized before being sonicated to reduce its size. Post homogenization, to vaporize the volatile dichloromethane layer, the mixture is stirred for 4–5 h using a continuous magnetic stirrer and then ultra-centrifuged at 17,000 rpm, 15 min, 4 °C. Clear supernatant is decanted, and the residue is resuspended in 2% *w*/*v* trehalose, which acts as a cryoprotectant, and kept in a Labconco™ benchtop freeze dryer. The lyophilization process was started, and after 72 h, a microparticulate powder form of vaccine was obtained. Characterization of microparticles was performed in the previous study [15].

Oral dissolving films (ODFs) were formulated using biodegradable polymers like Kollidon 90F (16.24% *w*/*v*) and Kollidon VA64 (1.06% *w*/*v*) and were made with ethanol as the solvent. Then, PEG 2000 (0.6% *w*/*v*), a film-forming polymer, was added to the prior mixture. This mixture was covered with foil and stirred for four hours and was kept standing overnight to remove trapped air. The mixture, along with vaccine microparticles, was put into the nozzle head of the CELLINK INKREDIBLE plus^®^ 3D bioprinter (Cellink, Gothenburg, Sweden). The ODF formulation was printed in a mold created in the lab. This mold was created using a SYLGARD 186 silicone elastomer kit (Dow Sylgard, Midland, MI, USA). The resulting mixture was poured into the mold and UV-crosslinked to form vaccine ODFs.

### 2.3. Characterization of Oral Dissolving Film

Oral dissolving films are tested for weight variation, uniform diameter, thickness, surface pH, and disintegration time. These tests are performed using *n* = 3 ODFs. Diameter and thickness were tested using Fisherbrand™ Traceable™ Digital Calipers (Thermo Fischer Scientific, Waltham, MA, USA). Diameter was analyzed from 4 different points, and the average was noted. To evaluate the disintegration time, each ODF is individually placed in a Petri plate, and the time is indicated when the ODF swells and disintegrates. PH is noted using the Thermo Scientific Orion Star™ A211 pH meter (Thermo Fisher Scientific, Waltham, MA, USA). Groups for all these tests include (1) Blank ODF (no vaccine particles), (2) WIV—Delta MPs loaded ODFs, (3) WIV—Omicron MPs + loaded ODFs, (4) WIV—Delta MPs + adjuvant loaded ODFs, (5) WIV—Omicron MPs + adjuvant loaded ODFs.

### 2.4. Analysis of Autophagosomes

Autophagy is an essential process for host defense, as it helps clear invading pathogens and supports antigen presentation, thereby strengthening the immune response. An autophagosome is an intermediate step in the dynamic process of autophagy. Autophagosomes formed after exposing the dendritic cells to different groups are analyzed using a fluorescence microscope (Lionheart FX, Biotek, VT, USA). Firstly, DCs are plated in a 48-well plate with a seeding density of 3 × 10^4^ cells per well. The next day, wells are exposed with different groups that include (1) Cells only, (2) Delta MPs + Adjuvant, (3) Omicron MPs + Adjuvant, and placed in an incubator overnight at 37 °C with 5% CO_2_. To detect autophagosomes, the CYTO-ID autophagy detection kit (Enzo Life Sciences, Farmingdale, NY, USA) was used, and the experiments were performed according to the manufacturer’s protocol. Cells were rinsed with phosphate-buffered saline (PBS), pH 7.4, and then stained with CYTO-ID^®^ dye and Hoechst 33342 Nuclear stain for 30 min at 37 °C. Following the incubation period, the unbound dye was rinsed, and the cell nucleus was stained with Hoechst 33342 Nuclear Stain, which appeared blue. Autophagosomes were labeled with CYTO-ID dye and appeared green. Strained cells were analyzed under a FITC filter and a DAPI filter for imaging.

### 2.5. In Vivo Administration

Six Swiss Webster female mice aged six to eight weeks per group were used in this study. A dose of 40 µg equivalent of antigen, along with 30 µg of Alum, was used. There are three groups: naïve (no treatment), intramuscular suspension (vaccine + adjuvant MPs), and ODFs microparticulate vaccine. A prime dose of whole cell-inactivated virus of Delta variant in suspension form was administered to mice intramuscularly. Buccal immunization was performed via microparticulate ODFs. Prime dose vaccination was carried out in the 2nd week, followed by a booster of Omicron variant in week 5, and euthanized in week 7. Blood and mucosal samples were obtained biweekly following each dose. Blood was withdrawn using the tail-snip procedure, and mucosal samples were taken through the vagina washes. Blood and vaginal samples were spun down at 13,000 RPM for 10 min at 4 °C, and the supernatant, which included the serum and mucosal samples, was stored at −80 °C until analyzed for antibodies.

### 2.6. Serum Antibodies and Mucosal Antibodies Evaluation Post-Immunization

Mucosal and serum antibodies formed post-immunization were analyzed using Enzyme linked immunosorbent assay (ELISA). In this study, mice serum was evaluated for IgM, IgA, IgG, and subtypes such as IgG1 and IgG2a against coated antigens such as SARS-CoV-2 variants Delta, Omicron, Alpha, and Gamma. On the first day, high-binding 96-well plates were coated with the coating antigen and kept at 4 °C for 24 h. The plate was cleaned with 0.05% Tween PBS the next day and blocked with 3% BSA for 3 h at 37 °C before adding mice serum samples that had been diluted 1:100 in 1% BSA. The plate is incubated for 24 h at 4 °C. Next, HRP-conjugated goat anti-mouse secondary antibody was added and incubated for 2 h. 3,3′,5,5′-tetramethylbenzidine (TMB) (BioLegend^®^, San Diego, CA, USA) was utilized as a substrate, with 0.3 M sulphuric acid used to stop the reaction. Plates were washed three times (0.1% Tween 20 in PBS) at each phase. The plate was measured at 450 nm with a BioTek^®^ Synergy H1 microplate reader (BIO-TEK Instruments, Winooski, VT, USA).

ELISA was also performed using the same process on lung supernatant samples. Post-sacrifice lung samples were collected and spun at 500× *g* for 15 min, 15 °C, and supernatant was collected, which was analyzed for antibodies such as IgM, IgG, and IgA.

### 2.7. Serum Neutralization Assay

Neutralization assay tells us whether antibodies generated after vaccination can actually neutralize the virus and are not just limited to the binding to it. To measure the levels of neutralization antibodies in the serum, the pseudovirus neutralization assay (PVNA) protocol was followed. Human epithelial kidney 293T human angiotensin converting enzyme 2 (HEK 293T-hACE 2) cells were grown and then plated in 6-well plate with a seeding density of 2 × 10^4^ cells/well overnight. The next day, in another 96-well round-bottom plate, serum dilutions (1:20 in DMEM 10%) of 30 μL/well were prepared, and 20 μL/well pseudovirus (1:500 in DMEM 10%) was added to it and incubated for 60–90 min at 37 °C on plate rotator. Media from the HEK 293T-hACE 2 plate was removed, and polybrene transfection reagent of 10 μL/well was added and the 50 μL/well mixture from the round-bottom flask was added to this plate and incubated for three hours at 37 °C, 5% CO_2_. Volume was made up to 150 μL followed by incubation of 48–60 h at 37 °C and 5% CO_2_. In the next step, medium was withdrawn, and cells were gently washed with phosphate-buffer saline. A total of 20 μL of 1× lysis buffer was added per well and gently shaken to mix. A total of 1× lysis buffer was made using Luciferase cell lysis 5× (E153A—Promega, Madison, WI, USA), wherein 1 part was combined with four parts of water. Later, the plate reader (Synergy H1 Biotek) was designed to do a 2 s measurement delay followed by a 10 s luminescence measurement. To perform the luciferase experiment, 100 μL of Luciferase assay reagent was made by mixing reconstituted Luciferase assay substrate (E151A—Promega, Madison, WI, USA) with 10 mL of Luciferase buffer (E152A—Promega, Madison, WI, USA) and was added to white lumitrac microplates (Greiner bio-one). The amount of 20 μL of cell lysate was pipetted into Lumitrac plates (Greiner Bio) and mixed 2–3 times. The plate was instantly read to determine the Relative Light Units (RLU). % Neutralization was calculated by subtracting the RLU values of the control group from the RLU values of serum samples, dividing by the RLU values of the control, and multiplying by 100. The values were plotted in the graphs.

### 2.8. Costimulatory Modulators

The upregulation of costimulatory molecules is a hallmark of dendritic cell activation and is essential for robust and durable immune responses, making it an important parameter to evaluate in vaccine research. Before investigating the immunogenicity of vaccine microparticles in vivo, we examined the vaccine’s antigen presentation in vitro using murine dendritic cells (DC 2.4). In a 48-well plate, DC 2.4 cells were seeded at a density of 30,000 cells per well and cultured overnight at 37 °C with 5% CO_2_. The following day, cells were pulsed with 200 μg of vaccine microparticles (1 mg/mL) per well. The pulsed cells were incubated at 37 °C with 5% CO_2_ for 24 h. Microparticle groups include (1) Blank microparticles, (2) Vaccine MPs, (3) Vaccine MPs with adjuvant, and (4) Cells only (control). Following incubation, the cells were washed with phosphate-buffered saline. The cells were extracted using a cell scraper and stained with fluorescein isothiocyanate (FITC)- and allophycocyanin (APC)-labeled anti-mouse MHC I, CD80, MHC II, and CD40 markers according to the manufacturer’s instructions (eBiosciences labs, San Diego, CA, USA; BioLegend, San Diego, CA, USA). Following one hour of incubation on ice, the cells were washed three times to eliminate any unbound markers. The cells were then suspended in phosphate-buffered saline, and the markers’ expression was measured using a BD Accuri C6 flow cytometer (BD Bioscience, San Jose, CA, USA).

### 2.9. CD4 and CD8a T-Cells Cellular Responses in Splenocytes

Analyzing CD4^+^ and CD8a^+^ T-cell responses provides essential insights into the durability of vaccine-induced immunity, making them critical endpoints in vaccine evaluation. After the mice were euthanized at terminal week, a critical immunological organ, such as the spleen, was taken to assess T cells. All organs obtained were separated into single-cell suspensions. All samples were passed through a 40 µm cell strainer and crushed using a plunger. To lyse red blood cells (RBCs) from the spleen samples, an ammonium chloride potassium (ACK) lysis buffer was used. The cells were then suspended in DMEM supplemented with 70% fetal bovine serum (FBS) and centrifuged at 1500 rpm for 10 min at 20 °C. Samples were kept at −80 °C with 5% *v*/*v* dimethyl sulfoxide (DMSO) for cryoprotection. We used a BD Accuri C6 Plus flow cytometer (BD Bioscience, San Jose, CA, USA) to analyze the samples and determine the expression of markers in splenocytes. On the first day of the process, approximately 100 μL of cells were withdrawn from the cell suspension and transferred to another Eppendorf tube. DMEM was added and the volume was made up to 500 μL, and the volume was made up to 500 μL. The cells were then placed in the incubator overnight. The next day, cells were stimulated with 5 µg/mL IL-2 and incubated overnight. The following day, cells were exposed to specific antigens and incubated for another 24 h. After stimulation, cells were centrifuged at 1200 rpm, discarded the supernatant, and resuspended in 100 μL of marker solution made by mixing anti-mouse APC-labeled CD4 and FITC-labeled CD8a markers in PBS and incubated on ice for 90 min, and vortexed every 15 min. Later on, cells were washed thrice with PBS, and flow cytometry was performed using BD Accuri C6 Plus Flow Cytometer (BD Bioscience, Ann Arbor, MI, USA).

### 2.10. Statistical Analysis

All statistical analyses were performed using the GraphPad Prism 10.4.1 program. Unless otherwise stated, all experiments were conducted in triplicate. Multiple group comparisons were conducted using two- and one-way analysis of variance (ANOVA), with post hoc analysis using Tukey’s and Dunnett’s multiple comparison tests. The data are presented as mean ± SEM. The *p* values used were as follows: *p* > 0.05 (non-significant), *p* ≤ 0.05 (*), *p* ≤ 0.01 (**), *p* ≤ 0.001 (***), and *p* ≤ 0.0001 (****).

## 3. Results

### 3.1. Characterization of Orally Dissolving Films

The results of prepared ODFs that were evaluated for physicochemical parameters, including average weight (mg), thickness (µm), diameter (mm), disintegration time (seconds), and pH, were summarized in Table 1. All six groups demonstrated consistent weights with no significant variation. The film thickness ranged from 0.110 to 0.175 µm, and the diameter was in the range of 0.168 to 0.172 µm. The overall release from the polymer was within 130 s. The pH values, ranging from 7.30 to 7.61, were well aligned with buccal mucosal conditions and are not harmful to the mucosal cavity.

### 3.2. Autophagy

**Figure 1:**
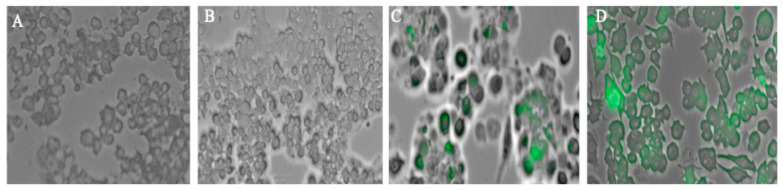
Autophagy in DCs treated with different groups (**A**–**D**). Fluorescent microscope imaging. (**A**) Cells only (−ve control). (**B**) Antigen suspension. (**C**) Vaccine MP. (**D**) Vaccine + Adjuvants (30 μg/mL) in MP. Except for the cells only group, 40 μg/mL of antigen was added to the other groups.

Autophagy is a highly regulated process that can be detected through measuring and visualizing the autophagosomes in cells responding to the induction by foreign particles. It plays a critical role in antigen presentation and thus is involved in immune response generation. In this study, the following four experimental groups were included: untreated dendritic cells (control), SARS-CoV-2 virus suspension (omicron variant) group, SARS-CoV-2 virus MPs (omicron variant) group, and adjuvanted SARS-CoV-2 MP. Autophagosomes were visualized using a green, fluorescent dye, GFP-LC3 puncta that is detected in the vesicles, which serves as a marker of autophagic activity. Among the groups, the adjuvanted MPs group and SARS-CoV-2 virus MPs significantly induced autophagosomes compared to the suspension group. Autophagy induction was quantified using a live cell imaging system (BioTek, Winooski, VT, USA), and the results are presented in Figure 1.

### 3.3. Serum and Mucosal Antibodies Post-Immunization and Neutralizing Antibody Levels in Serum

**Figure 2:**
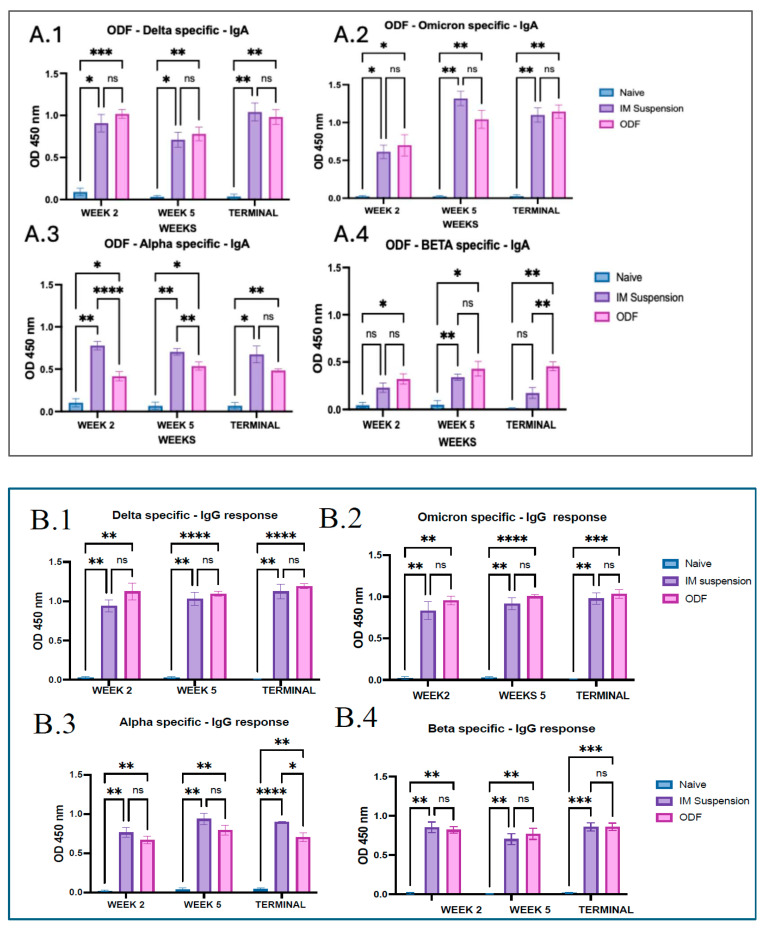

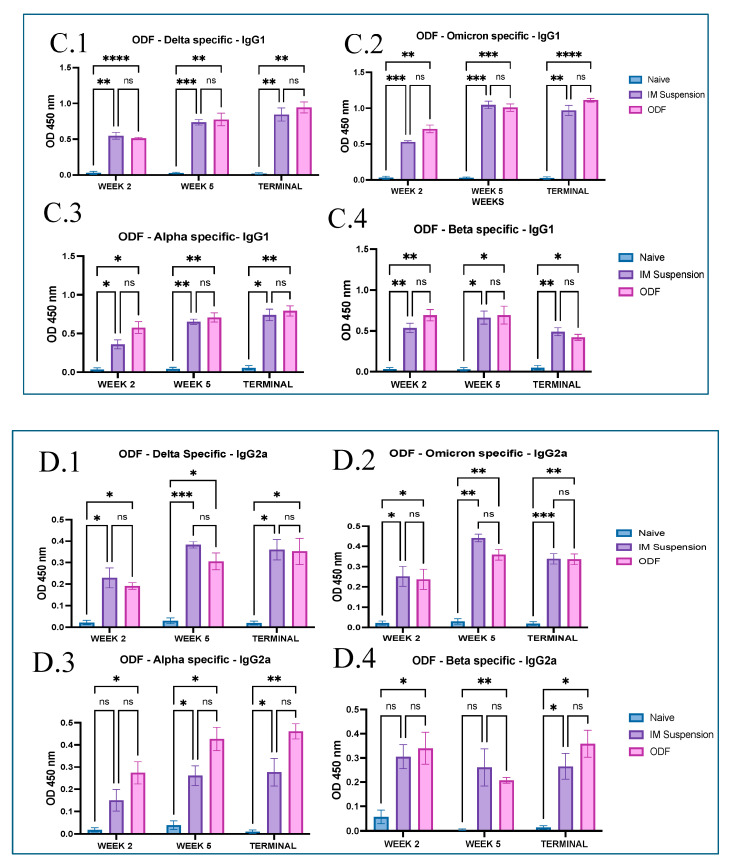
(**A**) Serum antibody levels in the serum of vaccinated mice. The serum antibodies were assessed against different antigens such as whole inactivated virus of Delta variant, Omicron variant, Alpha variant, Beta variant using ELISA for week 2, week 5, and the terminal week. (**A.1**–**A.4**) Antigen-specific IgA antibody response (**A.1**) represents Delta specific secretory IgA levels. (**A.2**) represents Omicron specific secretory IgA levels. (**A.3**) represents Alpha specific secretory IgA levels. (**A.4**) represents Beta specific secretory IgA levels. Responses obtained from the treatment group that is oral dissolving film (ODF) are compared to naïve (control) and intramuscular (IM) suspension group. Data are expressed as mean ± SEM, analyzed using two-way ANOVA followed by post hoc Tukey’s multiple comparison test—* *p* < 0.05, ** *p* < 0.01, *** *p* < 0.001, **** *p* < 0.0001, ns—non-significant. (**B**) Serum antibody levels in the serum of vaccinated mice. The serum antibodies were assessed against different antigens like whole inactivated virus of Delta variant, Omicron variant, Alpha variant, Beta variant using ELISA for week 2, week 5, and the terminal week. (**B.1**–**B.4**). Antigen-specific IgG antibody response. (**B.1**) represents Delta specific secretory IgG levels. (**B.2**) represents Omicron specific secretory IgG levels. (**B.3**) represents Alpha specific secretory IgG levels. (**B.4**) represents Beta specific secretory IgG levels. Responses obtained from the treatment group that is oral dissolving film (ODF) are compared to naïve (control) and intramuscular (IM) suspension group. Data are expressed as mean ± SEM, analyzed using two-way ANOVA, followed by post hoc Tukey’s multiple comparison test—* *p* < 0.05, ** *p* < 0.01, *** *p* < 0.001, **** *p* < 0.0001, ns—non-significant. (**C**) Serum antibody levels of vaccinated mice. The serum antibodies were assessed against whole inactivated virus of Delta variant, Omicron variant, Alpha variant, Beta variant using ELISA for week 2, week 5, and the terminal week. (**C.1**) represents Delta specific secretory IgG1 levels. (**C.2**) represents Omicron specific secretory IgG1 levels. (**C.3**) represents Alpha specific secretory IgG1 levels. (**C.4**) represents Beta specific secretory IgG1 levels. Responses obtained from the treatment group that is oral dissolving film (ODF) are compared to naïve (control) and intramuscular (IM) suspension group. Data are expressed as mean ± SEM, analyzed using two-way ANOVA followed by post hoc Tukey’s multiple comparison test—* *p* < 0.05, ** *p* < 0.01, *** *p* < 0.001, **** *p* < 0.0001, ns—non-significant. (**D**) Serum IgG2a antibody levels of vaccinated mice. The serum antibodies were assessed against whole inactivated virus of Delta variant, Omicron variant, Alpha variant, Beta variant using ELISA for week 2, week 5, and the terminal week. (**D.1**) represents Delta specific secretory IgG2a levels. (**D.2**) represents Omicron specific secretory IgG2a levels. (**D.3**) represents Alpha specific secretory IgG2a levels. (**D.4**) represents Beta specific secretory IgG2a levels. Responses obtained from the treatment group that is oral dissolving film (ODF) are compared to naïve (control) and intramuscular (IM) suspension group. Data are expressed as mean ± SEM, analyzed using two-way ANOVA followed by post hoc Tukey’s multiple comparison test—* *p* < 0.05, ** *p* < 0.01, *** *p* < 0.001, ns—non-significant.

Indirect ELISA was used to evaluate antibody levels in mouse serum samples. Mice that received both Delta and Omicron variant vaccines with adjuvant via oral dissolving films and intramuscular injections showed significantly elevated IgA responses specific to Delta and Omicron antigens at weeks 2, 5, and the terminal week compared to the untreated control group. In the Delta specific and omicron-specific IgM response, a particular trend of increase in immune response after a week was seen post-variant immunization. Alpha-specific and Beta-specific IgA levels were comparatively low but significant against the naïve group. Moreover, comparison between the intramuscular and buccal delivery groups revealed no significant differences in IgA responses against Delta, Omicron, and beta specific antibodies, except in terminal week where antibodies in ODF group were considerably higher than IM suspension group for Beta specific antibodies. In alpha specific response IgA antibodies in weeks 2 and 5 were significantly higher in intramuscular, which can be seen in Figure 2.

IgG antibody analysis demonstrated significantly elevated levels across all treatment groups compared to the untreated controls for all four SARS-CoV-2 variants. No notable differences were detected between the intramuscular and ODF administered groups during the study, with the exception of the Alpha variant at the terminal time point. To evaluate the type of immune response induced, IgG subtypes IgG1 and IgG2a—markers for Th2 and Th1 responses, respectively—were also assessed. An increasing trend was seen in IgG1 levels across the study for all antigens, except for Beta specific in the terminal week. IgG1 levels were significantly higher in the treatment groups relative to the untreated group for Delta, Omicron, and Alpha variants at all evaluated timepoints. A comparison between the microneedle and intramuscular delivery methods showed no significant difference for any strain except Delta. A similar pattern was observed with IgG2a levels for the ODFs group, showing significantly elevated antigen-specific responses throughout the study for all variants except Beta. There was no significance seen in week 2 and week 5 for the beta-specific IgG2a levels, and a similar result was observed at week 2 for alpha-specific IgG2a antibody. IgG2a levels were comparatively lower than IgG1 throughout the study for all four antigens, which can be seen in Figure 2.

**Figure 3:**
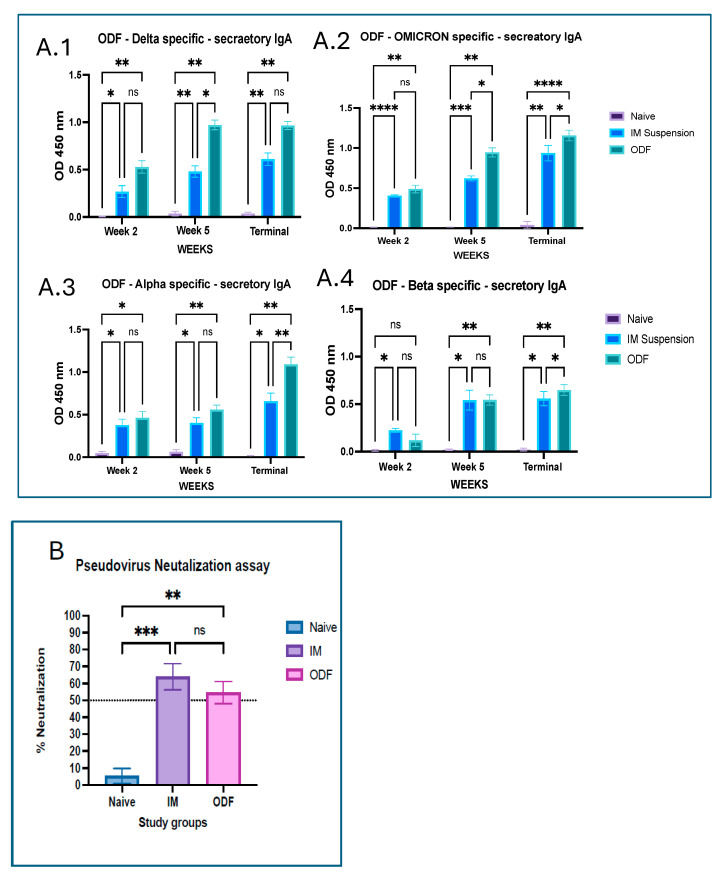
(**A**) 1–4 Mucosal IgA antibody levels in the vaginal washes of vaccinated mice using ELISA for week 2, 5, and the terminal week. (**A.1**) represents Delta specific secretory IgA levels. (**A.2**) represents Omicron specific secretory IgA levels. (**A.3**) represents Alpha specific secretory IgA levels. (**A.4**) represents Beta specific secretory IgA levels. Responses obtained from the treatment group that is oral dissolving film (ODF) are compared to naïve (control) and intramuscular (IM) suspension group. Data are expressed as mean ± SEM, analyzed using two-way ANOVA followed by post hoc Tukey’s multiple comparison test—* *p* < 0.05, ** *p* < 0.01, *** *p* < 0.001, **** *p* < 0.0001, ns—non-significant. (**B**) Pseudovirus neutralization assay performed for terminal week serum samples. Responses obtained from the treatment group that is oral dissolving film (ODF) are compared to naïve (control) and intramuscular (IM) suspension group. Data are expressed as mean ± SEM, analyzed using two-way ANOVA followed by post hoc Tukey’s multiple comparison test ** *p* < 0.01, *** *p* < 0.001, ns—non-significant.

Mice mucosal samples collected across all weeks were analyzed for secretory IgA levels; all four variants showed a significant increase in IgA levels in the treated groups compared to the naïve controls. Overall, there was an increasing trend seen across the study period in all antigens, wherein by the end of the terminal weeks, the maximum responses were seen. Moreover, mice vaccinated via the buccal route had significantly higher mucosal IgA levels as compared to the mice that received the intramuscular vaccine, which can be seen in Figure 3.

The functional activity of serum antibodies was further assessed by examining their capacity to neutralize the SARS-CoV-2 pseudovirus in the serum samples collected at the terminal week. Significant difference was observed between the naïve and treatment group, with neutralization as low as 2% in the naïve group. In the MPs’ ODF vaccine group, neutralization efficiency varied among animals, ranging from 48 to 60%, and in the vaccine IM MP suspension group, it was 55–70%; however, no significant difference was observed between the two groups.

### 3.4. Lung Supernatant Antibodies

**Figure 4:**
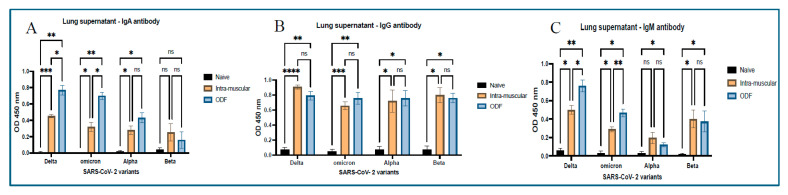
Mucosal antibody levels in the lung supernatants of vaccinated mice. These antibodies were assessed against different SARS-CoV-2 antigens for antibodies like IgM, IgG, and IgA using ELISA. (**A**) Antigen-specific IgA antibody response. (**B**) Antigen-specific IgG antibody response. (**C**) Antigen-specific IgM antibody response. Responses obtained are compared to naïve (control) and ODF MP suspension group. Data are expressed as mean ± SEM, analyzed using two-way ANOVA followed by post hoc Tukey’s multiple comparison test—* *p* < 0.05, ** *p* < 0.01, *** *p* < 0.001, **** *p* < 0.0001, ns—non-significant.

When an indirect ELISA was performed on the lung supernatant samples obtained from the mice, we could observe a significant increase in the IgA antibody levels across treatment groups for all antigens except the Beta-specific antigen. For IgG specific antibody, significance was seen in the treatment groups vs. the naïve group. No significance was seen between treatment groups across all antigens. After analyzing samples for IgM antibody, significant levels were observed for the treatment group vs. the no treatment group, except for alpha antigen; there was no significance seen for the intramuscular group vs. naïve group. In delta- and omicron-specific antigen analysis, there was significance seen within the treatment groups, which can be seen in Figure 4.

### 3.5. Costimulatory Modulators

**Figure 5:**
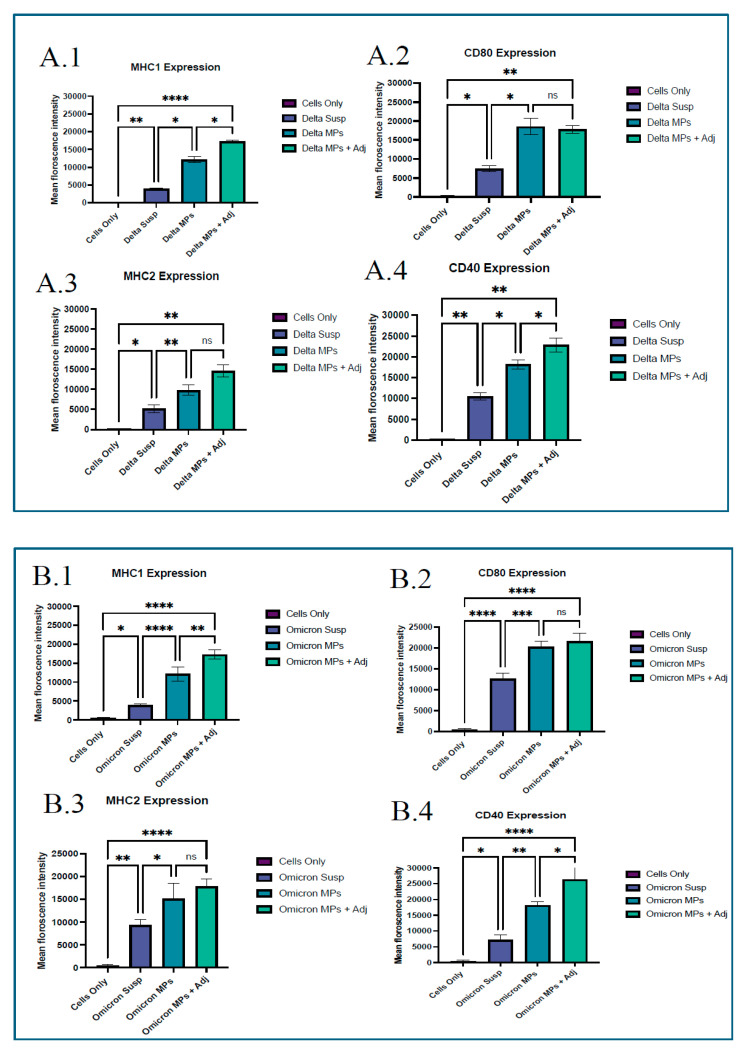
(**A**) Assessing costimulatory markers MHC1 and CD80, and MHC2 and CD40 in DCs exposed to whole inactivated virus of Delta variant using flow cytometry. (**A.1**) MHC1 expression in different groups (**A.2**) CD80 expression in different study groups. (**A.3**) MHC2 expression in the study groups (**A.4**) CD40 expression in study group. Responses obtained are compared to naïve (control) and Antigen (Delta variant) suspension groups and within the groups. Data are expressed as mean ± SEM, analyzed using two-way ANOVA followed by post hoc Tukey’s multiple comparison test—* *p* < 0.05, ** *p* < 0.01, **** *p* < 0.0001, ns—non-significant. (**B**) Assessing costimulatory markers MHC1 and CD80, and MHC2 and CD40 in DCs exposed to whole inactivated virus of Omicron variant exposed groups using flow (**B.1**) MHC1 expression in different groups. (**B.2**) CD80 expression in different study groups. (**B.3**) MHC2 expression in the study groups. (**B.4**) CD40 expression in study group. Responses obtained are compared to naïve (control) and Antigen (Delta/Omicron) suspension groups and within the groups. Data are expressed as mean ± SEM, analyzed using two-way ANOVA followed by post hoc Tukey’s multiple comparison test—* *p* < 0.05, ** *p* < 0.01, *** *p* < 0.001, **** *p* < 0.0001, ns—non-significant.

The results indicate a significant upregulation of MHC class I and II surface expression on dendritic cells treated with the adjuvanted vaccine microparticles, compared to those exposed to whole-cell inactivated SARS-CoV-2 variants, that is, Delta and Omicron in suspension form without microparticles. Similarly, costimulatory markers CD40 and CD80 were markedly elevated in the adjuvanted MP-treated group compared to the untreated control. In contrast, the untreated DC 2.4 control group failed to show any induction of costimulatory molecule expression. Suspension group showed significance against naïve group; however, lower levels were observed against the microparticulate group along with the adjuvant group, which can be seen in Figure 5.

### 3.6. Cellular Response

**Figure 6:**
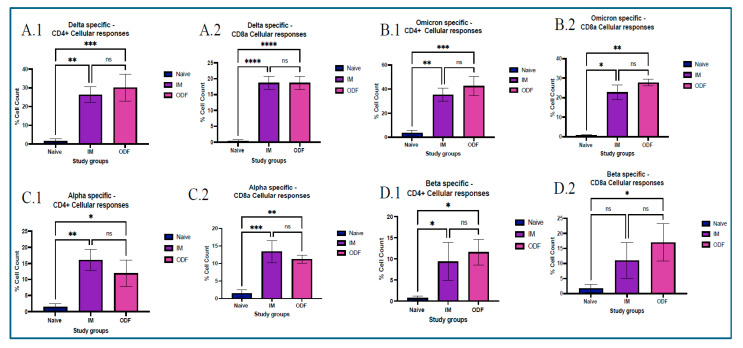
Assessing CD4^+^ and CD8a cellular markers in Spleenocytes exposed to Delta, Omicron, Alpha, Beta antigens and analyzed using flow cytometry. (**A**) 1,2 Delta-specific marker response. (**A.1**) represents Delta CD4^+^ cellular responses, (**A.2**) represents Delta CD8a cellular responses (**B**) 1,2. Omicron-specific marker response, (**B.1**) represents Delta CD4^+^ cellular responses, (**B.2**) represents Delta CD8a cellular responses (**C**) 1,2 Alpha-specific marker response. (**C.1**) represents Delta CD4^+^ cellular responses, (**C.2**) represents Delta CD8a cellular responses (**D**) 1,2 Beta-specific marker response. (**D.1**) represents Delta specific CD4^+^ cellular responses, (**D.2**) represents Delta CD8a cellular responses. Responses obtained are compared to naïve (control) and IM MN group. Data are expressed as mean ± SEM, analyzed using two-way ANOVA followed by post hoc Tukey’s multiple comparison test—* *p* < 0.05, ** *p* < 0.01, *** *p* < 0.001, **** *p* < 0.0001, ns—non-significant.

We utilized flow cytometry to assess CD4^+^ and CD8a T-cell populations within splenocytes and lymph nodes of vaccinated mice. Mice immunized with the ODF microparticulate vaccine exhibited significantly elevated CD4^+^ T-cell and CD8a T-cell responses in splenocytes compared to the untreated control group across all antigens. However, CD8+ T-cell populations across the Beta specific antigen were not significant for the IM suspension group when compared to the naïve group. Additionally, levels of antigen-specific CD8a T-cell responses were lower than those of CD4^+^ T-cells in Delta- and Omicron-specific antigens. The magnitude of CD8a T-cell activation following ODF vaccination was on par with that of the IM suspension group, which can be seen in Figure 6.

**Figure 7:**
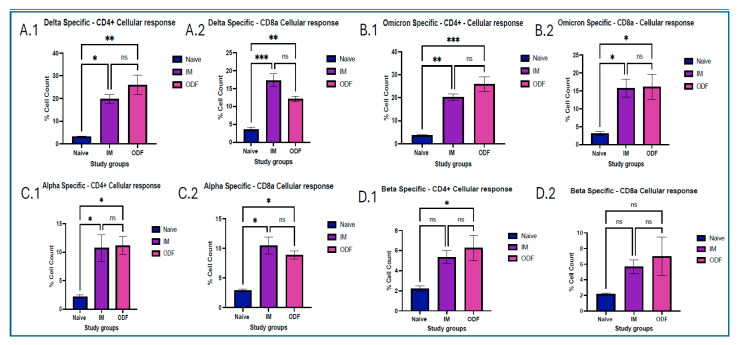
Assessing CD4^+^ and CD8a cellular markers in lymph nodes exposed to Delta, Omicron, Alpha, Beta antigens and analyzed using flow cytometry. (**A**) 1,2 Delta-specific marker response. (**A.1**) represents Delta CD4^+^ cellular responses, (**A.2**) represents Delta CD8a cellular responses (**B**) 1,2.Omicron-specific marker response, (**B.1**) represents Delta CD4^+^ cellular responses, (**B.2**) represents Delta CD8a cellular responses (**C**) 1,2 Alpha-specific marker response. (**C.1**) represents Delta CD4^+^ cellular responses, (**C.2**) represents Delta CD8a cellular responses (**D**) 1,2 Beta-specific marker response. (**D.1**) represents Delta specific CD4^+^ cellular responses, (**D.2**) represents Delta CD8a cellular responses. Responses obtained are compared to naïve (control) and IM MN group. Data are expressed as mean ± SEM, analyzed using two-way ANOVA followed by post hoc Tukey’s multiple comparison test—* *p* < 0.05, ** *p* < 0.01, *** *p* < 0.001, ns—non-significant.

Mice immunized with the ODF microparticulate vaccine exhibited significantly elevated CD4^+^ T-cell and CD8a T-cell responses in lymph node samples compared to the untreated control group across all antigens. However, CD8a T-cell populations across the Beta specific antigen were not significant for the treatment groups when compared to the naïve group. Additionally, levels of antigen-specific CD8a T-cell responses were lower than those of CD4^+^ T-cells in Delta- and Omicron-specific antigens. There was no significant difference in the antigen specific CD8a and CD4^+^ cellular responses between the groups receiving intramuscular suspension and oral dissolving films, which can be seen in Figure 7.

## 4. Discussion

The study aimed to create a whole cell inactivated SARS-CoV-2 microparticulate-based vaccine which is further formulated into oral dissolving films. To assess the formulation’s capacity to elicit immune response, microparticulate ODFs were delivered to mice. For the successful delivery of oral dissolving films through the buccal route, characterization of ODFs plays a vital role [21]. The characterization results showed that the pH of the ODFs is in a non-irritating range, with adequate disintegration time of approximately 2 min, thickness, and diameter within 0.175 µm suitable enough to fit in the cheek of the mice. This means that ODFs will be delivered effectively causing minimal discomfort.

Autophagosomes are specialized vesicular structures formed during the process of autophagy and play a crucial role in antigen processing for presentation on both MHC class I and II molecules. After encountering a pathogen, innate antigen-presenting cells (APCs), primarily dendritic cells, macrophages, and B cells, internalize and process pathogen-derived proteins. These proteins (antigens) are then displayed on the cell surface bound to major histocompatibility complex (MHC) molecules (MHC I or II). This presentation is recognized by T-cell receptors (TCRs) on naïve adaptive immune cells after signaling of the costimulatory response [22]. Autophagy in DCs facilitates the delivery of cytoplasmic and phagocytosed antigens to MHC class II compartments, promoting CD4^+^ T cell activation. It also supports cross-presentation mechanisms that load antigens onto MHC class I molecules, crucial for CD8a T cell activation. Experimental studies show that autophagy gene deficiency in DCs reduces their ability to activate CD4^+^ T cells and impairs immune responses, underlining autophagy’s importance [23,24,25]. Complete activation of T-cells is possible after activating costimulatory molecules that provide a second signal, in conjunction with antigen recognition by the T cell receptor (TCR). Without co-stimulation, T cells recognizing an antigen may become anergic (non-responsive) or undergo apoptosis, preventing an effective immune response [26]. The SARS-CoV-2 microparticles demonstrated autophagosome formation when taken up by antigen-presenting dendritic cells. Autophagosomes play a pivotal role in bridging immune surveillance, especially in the context of antigen presentation [27]. The successful activation of costimulatory molecules CD80 and CD40, and antigen-presenting molecules MHCI, MHCII was observed in the study group; however, it was high in the Delta MPs + alum MP and Omicron MPs + alum group.

Mucosal immunization is an excellent approach for respiratory viruses like SARS-CoV-2 as it induces both local mucosal immunity at the site of viral entry, that is, the respiratory tract and stimulates immune responses directly in these mucosal tissues, establishing the first line of defense. Moreover, generation of systemic response offers more effective and comprehensive protection than traditional intramuscular vaccines [28]. We analyzed secretory IgA, generated at the localized mucosal sites from the collected vaginal samples. Significantly higher antibody levels were observed in the mice groups that received ODFs than in the mice that received intramuscular vaccine injections. Moreover, antibodies analyzed from lung supernatant samples show higher IgA antibody levels in ODF groups than in IM groups. This indicates buccal route could generate stronger localized immune response as compared to the intramuscular route. In addition to the IgA antibody, significant IgG and IgM antibody response was also observed in the lung supernatant samples, denoting the generation of systemic immune response along with mucosal immune response. The antibody response observed across the model antigen was more evident than that seen against the cross-reactive antigens, such as Alpha and Beta strain, due to antigen-specific immunity being generated post-administration.

Serum antibodies are generated when activated helper T cells stimulate B cells that recognize the antigen. Some B cells, upon stimulation, become plasma cells that migrate to systemic sites and produce antibodies, which enter the bloodstream as serum antibodies [29]. IgG antibody is the most abundant antibody in serum and critical for long-term immunity. It is the primary antibody responsible for neutralizing pathogens, opsonization, and activating complement pathways [30,31]. We have observed increased levels of IgG throughout the study period for treatment groups, which means initiation of the humoral immune response. Moreover, subtypes of IgG, such as IgG1 and IgG2a, indicated Th2 and Th1 immune responses, respectively [32]. Levels for mice having Omicron- and delta-specific antibodies were significantly higher in the treatment group against the IgG2a antibody, which suggests a Th2-biased immune response; however, the Th1 response was significantly against the no-treatment groups. Serum IgA responses are increasingly recognized in vaccines for respiratory viruses and play an important role in early neutralization [33]. We have observed increased levels of serum IgA throughout the study period for mice vaccinated with omicron and delta MPs. Furthermore, a significant cross-reactive immune response was observed for all antibodies. However, serum IgA levels were significantly low for Beta-specific antibody. This may be due to the differences in variants’ mutations, especially in the receptor-binding domain and key antigenic sites, causing antigenic drift, making these regions less easily recognized by antibodies. In addition, the deletion of residues L242–244 and the T95I mutation in the N-terminal domain altered its structural conformation, distinguishing Beta from variants such as Omicron and Delta and further decreasing antibody binding efficiency [34]. Mutations like N501Y in the receptor-binding domain and P681H mutation at the spike are major ones in the alpha variant, which enhance ACE2 binding and aid immune evasion [2]. We also quantified functional antibodies present in the terminal week serum samples using a pseudovirus neutralization assay [35]. This assay is considered a safe and practical alternative to traditional challenge studies that involve exposure to live pathogenic viruses. Neutralizing antibodies measured by this assay closely correlate with protection against COVID-19; higher neutralizing titers generally indicate greater vaccine efficacy [36,37]. As we could see more than 50% neutralization in the terminal week, it is inferred that our in-house vaccine particles produced functional antibodies that can protect the immune system and fight against the antigen on exposure to live virus.

Antigen presentation from MHC class of molecules leads to functions that promote proliferation, cytokine secretion, and cytotoxic effect. This occurs due to activation of a helper or cytotoxic T cells. CD4^+^ helper T cells become activated after displaying antigens on MHC class II molecules, showing a Th2 response, and presentation of antigens on MHC class I molecules activates CD8a cytotoxic T cells, showing a Th1 response. This can be identified by CD4^+^ and CD8a markers, respectively [32,38,39]. We found that mice vaccinated with Delta MPs + Alum MPs, and Omicron MPs + Alum MPs induced significant levels of helper T cells and cytotoxic T cells. This validated the presence of Th1 and Th2 immune responses in mice vaccinated. Moreover, the level of CD4 marker was higher than CD8a, showing a Th2 bias nature, thus indicating more cytokine secretion and further immune activation.

In this study, we have also used a heterologous approach. In previous studies, researchers have observed higher and sustained effectiveness with tolerable safety profiles and better neutralization antibodies [40,41]. Similarly, we observed elevated IgG and IgA responses, as well as strong neutralizing antibody titers and robust T-cell activation, indicating not only antigen-specific immunity but also broader cross-reactive immunity. Our heterologous delivery of microparticulate oral dissolving film indicated the potential to generate cross-reactive humoral and cellular response. Moreover, the systemic immune response generated by administrating ODFs is comparable to the response produced by painful intramuscular delivery of microparticulate vaccine, indicating the effective delivery through non-invasive route.

## 5. Conclusions

In this proof-of-concept work, we successfully developed an adjuvanted microparticle-based vaccine incorporated into oral dissolving films and administered it via the buccal route and analyzed humoral and cellular immune responses in the murine models. Our in vitro autophagy assay demonstrates the formation of autophagosomes, which is a process of antigen presentation. In vivo evaluations revealed that vaccination elicited robust systemic and mucosal immunity in mucosal samples collected from vaginal fluids and lung supernatants in mice. In addition, to determine if the antibodies were functional, we evaluated the neutralizing nature of the antibodies present in the serum samples. Moreover, cellular T-cell responses CD4^+^ and CD8a responses were observed in the spleen samples, demonstrating the potential for cytotoxicity and potent antibody and memory responses. Notably, the microparticulate heterologous vaccine also generated cross-reactive antibody responses in mice serum samples and secretory IgA antibody and CD4^+^ and CD8a T cell responses against the SARS-CoV-2 Alpha and Beta variants. In our further studies, we plan to evaluate the memory markers and cytokines present in the organ samples.

## Figures and Tables

**Table 1:** Characterization of oral dissolving films (ODFs) were performed. Different ODFs groups comprise Blank Oral dissolving films (ODFs), adjuvant based ODF, Delta + adjuvant (adj) suspension based ODF, Delta + Adjuvant (adj) microparticle ODF, Omicron + adjuvant (adj) suspension based ODF, Omicron + Adjuvant (adj) microparticle ODF were tested across different parameters for weight variation, diameter, thickness, surface pH, disintegration time. Data are expressed as mean ± SD.

Parameters	Blank ODFs	Adjuvants	Delta + Adjuvant Susp ODFs	Delta + Adjuvant MPs ODFs	Omicron + Adjuvant Suspension	Omicron + Adjuvant MPs ODFs
Weight Variation (mg)	9.1± 0.88	9.3 ± 0.47	9.5 ± 0.29	9.8 ± 0.73	9.4 ± 0.58	9.7 ± 1.04
Diameter (mm)	0.169 ± 0.02	0.168 ± 0.11	0.172 ± 0.07	0.169 ± 0.22	0.170 ± 0.17	0.172 ± 0.08
Thickness (µm)	0.115 ± 0.01	0.148 ± 0.01	0.166 ± 0.01	0.160 ± 0.01	0.174 ± 0.01	0.170 ± 0.01
Surface pH	7.3 ± 0.07	7.50 ± 0.05	7.50 ± 0.03	7.54 ± 0.04	7.61 ± 0.05	7.53 ± 0.03
Disintegartion Time (s)	98 ± 3.67	120 ± 2.13	124 ± 1.34	127 ± 3.82	101 ± 4.22	123 ± 2.56

## Data Availability

The data presented in this study are available upon request from the corresponding author.
